# Perioperative evaluation of tumescent anaesthesia technique in bitches submitted to unilateral mastectomy

**DOI:** 10.1186/1746-6148-9-178

**Published:** 2013-09-11

**Authors:** Leonardo de Freitas Guimaraes Arcoverde Credie, Stelio Pacca Loureiro Luna, Fabio Futema, Luciano Cacciari Baruffaldi Almeida da Silva, Giancarlo Bressane Gomes, Jaqueline Neratika Negrette Garcia, Lidia Raquel de Carvalho

**Affiliations:** 1 Department of Anaesthesiology, Faculty of Medicine, UNESP – Univ Estadual Paulista, Botucatu, Sao Paulo, Brazil; 2 Department of Veterinary Surgery and Anaesthesiology, Faculty of Veterinary Medicine and Animal Science, UNESP – Univ Estadual Paulista, Botucatu, Sao Paulo, Brazil; 3 University of Guarulhos, Guarulhos, Sao Paulo, Brazil; 4 Department of Biostathistics, Biosciences Institute, UNESP – Univ Estadual Paulista, Botucatu, Sao Paulo, Brazil

**Keywords:** Dogs, Mastectomy, Regional anaesthesia, Lidocaine

## Abstract

**Background:**

Tumescent anaesthesia (TA) is a widely used technique in oncologic surgeries necessitating large resection margins. This technique produces transoperative and postoperative analgesia, reduces surgical bleeding, and facilitates tissue divulsion. This prospective, randomised, blind study evaluated the use of TA in bitches submitted to mastectomy and compared the effect of TA with an intravenous fentanyl bolus. A 2.5-mcg/kg intravenous fentanyl bolus (n = 10) was compared with TA using 0.275% lidocaine (n = 10) in bitches submitted to unilateral mastectomy. Sedation was performed by intramuscular (IM) injection of 0.05 mg/kg of acepromazine combined with 2 mg/kg of meperidine. Anaesthesia was induced with 5 mg/kg of intravenous propofol and maintained with isoflurane/O_2_. Heart and respiratory rates; systolic, mean, and diastolic arterial blood pressures; central venous pressure; SpO_2_; ETCO_2_; inspired and expired isoflurane concentrations; and temperature were measured transoperatively. Visual analogue scales for sedation and pain and the Glasgow composite and Melbourne pain scales were used for postoperative assessment. The surgeon investigated the quality of the surgical approach, considering bleeding and resection ability, and the incidence of postoperative wound complications.

**Results:**

The heart rate was lower and the end-tidal isoflurane concentration was higher in dogs treated with fentanyl than in dogs treated with TA. A fentanyl bolus was administered to 8 of 10 dogs treated with fentanyl and to none treated with TA. Intraoperative bleeding and the mammary gland excision time were lower in dogs treated with TA. The maximal mean and individual plasma lidocaine concentrations were 1426 ± 502 ng/ml and 2443 ng/ml at 90 minutes after infiltration, respectively. The Glasgow Composite Pain Scale scores were higher in dogs treated with fentanyl than in dogs treated with TA until 2 hours after extubation.

**Conclusions:**

Compared with intravenous fentanyl, TA in bitches: may be easily performed in non-inflamed, ulcerated, adhered mammary tumours; has an isoflurane-sparing effect; improves transoperative and immediate postoperative analgesia; is apparently safe for use in clinical conditions as evidenced by the fact that it did not produce any adverse signs or lidocaine plasma concentrations compatible with toxicity; does not modify the recovery time; and facilitates the surgical procedure without interfering with wound healing.

## Background

Mammary tumours are the most common neoplasia in dogs, and mastectomy is the most indicated treatment with the exception of cases of inflammatory carcinoma and metastases [1]. Mastectomy requires extensive tissue resection to achieve a safe, wide margin and avoid recurrence. Under these circumstances, inappropriate anaesthetic and analgesic techniques may result in acute and chronic postoperative pain [2]. Thus, effective transoperative and postoperative analgesia is required.

Complex surgeries require multimodal analgesia, which is achieved by the combination of peripheral and central analgesic agents, to produce analgesic synergism and reduce adverse effects [3]. Regional analgesia, which is part of multimodal analgesia, reduces the perioperative requirement of anaesthetic and analgesic drugs and improves postanaesthetic recovery by inhibition of nociception [4].

The perioperative use of opioids is a traditional practice in oncology [5]. However, undesirable effects of pure opioid agonists of particular concern in oncology patients have been reported, such as immunosuppression, stimulation of neoplastic cell growth, and increased risk of tumour metastases [6-9].

Tumescent anaesthesia (TA) is a local anaesthetic technique used for infiltration of large areas of the body using large volumes of diluted local anaesthetic solution, usually combined with vasoconstrictors [10]. In this context, this technique appears to be promising for regional anaesthesia in cases of mammectomy. Considering the diluted nature of the solution, large amounts of anaesthetic solution may be safely used with little risk of local anaesthetic-induced toxicity [11]. In addition to providing analgesia, this technique reduces intraoperative bleeding [12].

The first local TA solution was reported by Klein in 1987 [13]. Since then, however, the composition has been modified from that described in the original report, mostly by changing the lidocaine and adrenaline concentrations. TA has been used for several procedures in human medicine, such as liposuction [13,14]; dermatological [15], mammary [16,17], facial plastic [18], and vascular [19] surgeries; sentinel node biopsy in melanoma [20]; massive resections [21]; and surgical treatment of burns and burn sequelae in children [22].

The main reported advantages of TA include intraoperative [23] and postoperative analgesia [21,23], better intraoperative haemostasis [14,21], decreased surgical cost [23], and hydrodissection of tissues, which facilitates surgery and minimises surgical trauma [21].

A rare reported complication of TA is pulmonary oedema. Thus, care should be taken to avoid fluid overload [10,24]. Care should also be taken with the concomitant chronic use of drugs that may slow lidocaine biotransformation and increase lidocaine-induced toxicity [25] by inhibiting the cytochrome P-450 isoenzymes CYP3A4 and CYP1A2, such as benzodiazepines, metronidazole, amiodarone, omeprazole, and verapamil. Other possible complications of TA are local anaesthetic intravascular injection, increased lidocaine toxicity by the concomitant use of sedatives [10,12], peri-incisional tissue oedema, risk of seeding neoplastic cells throughout the surgical site by the mechanical action of the cannula, and risk of further contamination in cases of skin infection or ulcers present in the tumours; TA is not recommended under this last circumstance.

Use of the Klein cannula is indicated for injection of large volumes of solutions. Its large diameter and presence of several lateral holes provide greater dispersion of the solution. The blunt tip also minimises vascular lesions, thoracic and abdominal penetration, and pain from injection [26]. A modified closed circuit can be used for infiltration [27], reducing both the time to perform the technique and the risk of infection.

To our knowledge, there is no report of the use of TA in veterinary medicine. The hypothesis of this study was that TA produces effective perioperative analgesia with apparent low toxicity and facilitates and reduces the mammary gland excision time during mastectomy in bitches.

The aim of this study was to compare the perianaesthetic and surgical effects of the use of TA in bitches submitted to unilateral mastectomy against intravenous (IV) administration of fentanyl.

## Methods

This study was previously approved by the Ethical Committee on the Use of Animals for Research and Teaching from the Faculty of Veterinary Medicine and Animal Science, UNESP, Botucatu, Sao Paulo, Brazil (protocol number 137/2010). After obtaining the owners’ consent, 20 bitches of different breeds ranging from 5 to 13 years of age, weighing 13.57 ± 9.57 kg, and classified as ASA I or II were submitted to unilateral mastectomy for treatment of mammary tumours. Selection criteria were based on normal complete blood test results, renal and hepatic function test results, and radiography and ultrasonography findings. Animals with metastases; tumours in other systems; inflamed, ulcerated, or adhered mammary tumours; and tumours of >5 cm in diameter were excluded.

Water and food were withheld for 6 and 12 hours, respectively, prior to surgery. Sedation was performed with 0.05 mg/kg of acepromazine^a^ combined with 2 mg/kg of meperidine^b^ intramuscularly. An IV catheter was placed in the right cephalic vein for administration of saline solution at 20 ml/kg/h. Another catheter was introduced into the left cephalic vein for collection of blood samples to analyse plasma lidocaine concentrations. Central venous pressure (CVP) was measured through a CVP catheter inserted in the right jugular vein. Anaesthesia was induced with 5 mg/kg of propofol^c^ IV followed by endotracheal intubation and was maintained with isoflurane^d^/O_2_ using a calibrated vaporiser^e^ and a rebreathing or non-rebreathing circuit according to the weight of the animal (> 7 or < 7 kg, respectively). All animals were maintained under spontaneous respiration. After 15 minutes of stabilisation of anaesthesia and monitoring, the animals were divided in two groups using a randomised, blind protocol.

**Figure 1:**
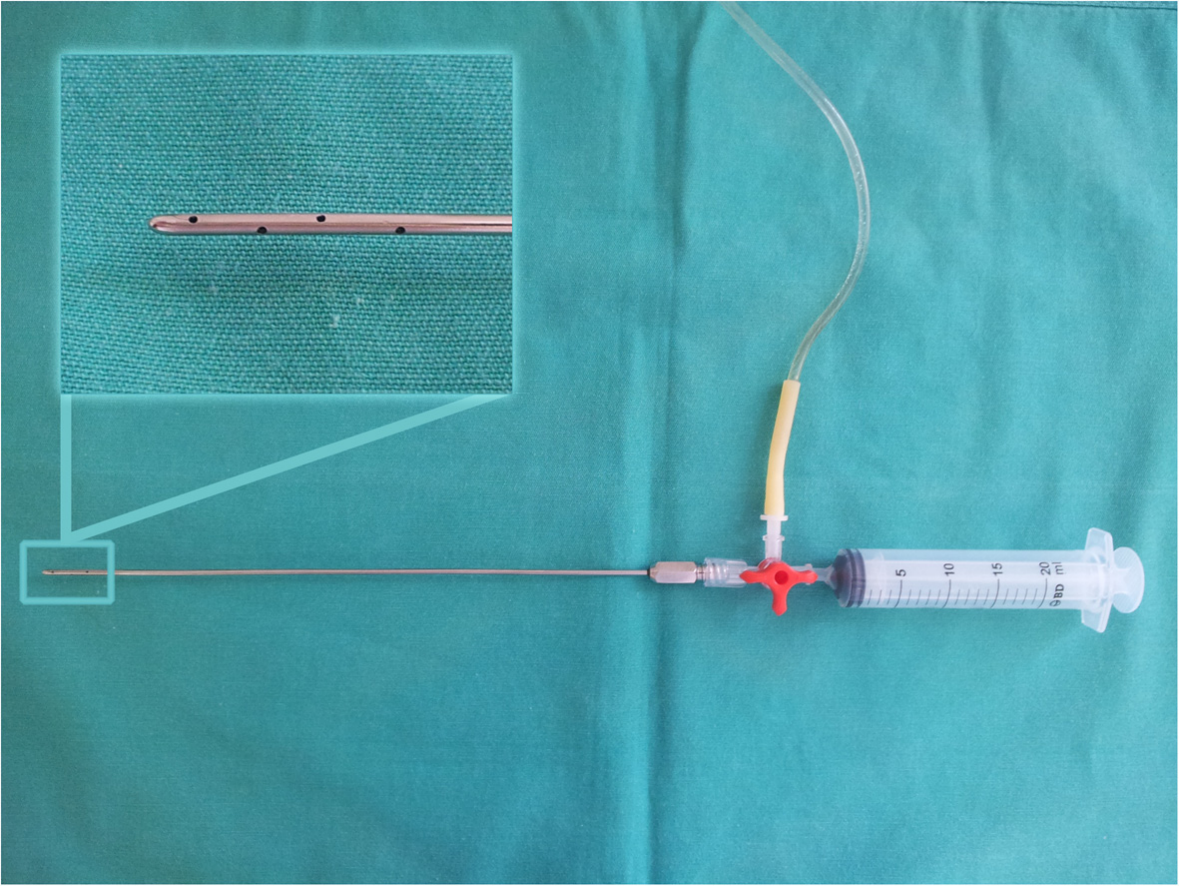
Closed circuit used to infiltrate the tumescent solution with the detail of the blunt infiltration cannula with side holes.

Animals were treated with either TA (GT) or IV fentanyl^f^ (GF). TA was performed by introduction of a Klein cannula (Figure 1) after creation of a small skin incision. The local anaesthetic solution (15 ml/kg at 4°C) was injected under extension of the target mammary glands. The tumescent solution was prepared with 250 ml of lactated Ringer’s solution, 40 ml of 2% lidocaine, and 0.29 ml of adrenaline (1 mg/ml) to achieve a lidocaine concentration of 2.75 mg/ml (0.275%) and total lidocaine dose of 41.25 mg/kg. The solution bag was connected through a three-way tap to a 20-ml syringe in one end and the Klein cannula in the other end to create a closed system (Figure 1).

TA was performed with the animal in dorsal recumbency, starting in the thoracic region, moving to the abdominal region, and finishing in the inguinal mammae (Additional file 1: Video 1). The infusion of the solution was concomitant with the advancement of the cannula. Two points of insertion were used: immediately cranial and caudal to the thoracic and inguinal mammae, respectively, avoiding the pudendal vessels in the inguinal region.

Animals in the GF group received a bolus of 2.5 mcg/kg of fentanyl IV, diluted to 0.005 mg/ml. Injection was performed slowly to avoid bradycardia or respiratory depression, and the injection speed was based on changes in the respiratory and heart rates and end-tidal carbon dioxide concentration (ETCO_2_).

After one of the analgesic techniques had been performed, a drape was placed in the vertical direction at the cervical region of the animal, to isolate the head and monitoring equipment from the surgical area and avoid visual contact of the anaesthetist with the surgical field. Next, a second anaesthetist (blind) was called to conduct the anaesthetic procedure. Surgery was performed by the same experienced surgeon (not blind) in all cases and started 10 minutes after analgesia had been completed in each group. The isoflurane concentration was adjusted to provide a sufficient depth of anaesthesia as judged by the ocular reflexes and increase in blood pressure and heart and respiratory rates above 20% when compared with preoperative values. However, when these changes were observed in direct association with the surgical stimulus, even when an adequate depth of anaesthesia was present, the anaesthetist (blind) was allowed to use IV fentanyl whenever necessary.

The heart and respiratory rates and temperature were measured before, during, and after anaesthesia, and invasive arterial blood pressure, CVP, oxygen haemoglobin saturation (SpO_2_), ETCO_2_, and inspired and end-tidal (ETIso) isoflurane concentrations were measured during anaesthesia using a multiparametric monitor and gas analyser^g^ at the following time points: 5 minutes after analgesia (PA), at the beginning of the first incision (I); thoracic (TM), abdominal (AM), and inguinal mammary gland divulsion (IM); pudendal artery and vein clamping (PAV) and surgical synthesis (S); 15 minutes after surgical synthesis (S15); and immediately after the end of surgery (ES). Anaesthetic, recovery, and surgical times were recorded.

After the end of the surgery, 0.1 mg/kg of meloxicam was injected subcutaneously, and the surgeon classified bleeding and facility to remove the mammary gland as increased (3), normal (2), or reduced (1) and difficult (3), normal (2), or easy (1), respectively.

In animals in the GT group, blood samples were collected at 10, 30, and 90 minutes and at 3 and 6 hours after TA in lithium heparin tubes for measurement of the plasma lidocaine concentration. The blood was centrifuged and the plasma was harvested and stored at −80°C. The lidocaine concentration was measured by high-performance liquid chromatography coupled with mass spectrometry with positive electrospray ionisation, monitoring the ions resulting from lidocaine fragmentation. The assay limit of detection (sensitivity) was 0.1 ng/ml, the inter-assay coefficient of variation was 2.05%, and the recovery rate was 96.5%.

Postoperative pain was investigated using the dynamic and interactive visual analogue scale (DIVAS), modified Glasgow Composite Pain Scale (GCPS), University of Melbourne Pain Scale (UMPS), and von Frey filament test at extubation; 30, 60, 120, 240, and 360 minutes after extubation; and 2, 5, and approximately 14 days after surgery. The surgical wound was also evaluated at the same time points. Tumour recurrence was followed every 3 months by phone calls. When the owner reported any problem, the animals were brought to the veterinary hospital for detailed evaluation.

Sedation was investigated at the same time points using a visual analogue scale (VAS). Mammary tumour histopathological examination was performed in all but one case.

Intraoperative analgesia was accomplished with 2.5 mcg/kg of fentanyl IV when the systolic/mean arterial blood pressure or heart rate increased above 20% of the postanalgesia value (PA). Postoperative analgesia was accomplished with 0.5 mg/kg of IM morphine^h^ when the GCPS score was > 3.33 and the sedation score was < 50. A new analgesic rescue was performed using the same dose of morphine when the GCPS score was not reduced after 30 minutes following the first analgesic rescue or when the DIVAS, GCPS, or UMPS score was > 33, > 5 or > 9, respectively.

Statistical analysis was performed using Student’s *t* test to compare age, weight, and anaesthetic, surgical, and recovery times. Analysis of variance was used to investigate differences in parametric variables, followed by Bonferroni’s or Tukey’s test to investigate differences in time in each group and between groups respectively (intraoperative cardiorespiratory data). The Friedman and Mann–Whitney tests were used for non-parametric variables to compare differences in time in each group and differences between groups, respectively (postoperative pain and sedation data). The chi square and Fisher tests were used for frequency analysis (bleeding and surgical ease). The level of significance was 5%.

## Results and discussion

The intraoperative results are presented as mean and standard deviation, followed by minimum and maximal values. The heart rate during the surgical procedure was significantly higher in the GT group until inguinal mammary gland divulsion (111 ± 16 beats/min; range, 86–160 beats/min) than in the GF group (94 ± 19 beats/min; range, 38–140 beats/min) during anaesthesia. Bradycardia (heart rate of <60 beats/min) associated with second-degree atrioventricular blockade occurred in three animals in the GF group, but only one animal required atropine. This is a typical effect observed with the use of fentanyl and has been previously reported [28].

There were no differences in systolic (SABP), mean (MABP), and diastolic (DABP) arterial blood pressures either between groups (SABP: GT, 94 ± 21, 61–166; GF, 98 ± 15, 63–160; MABP: GT, 71 ± 13, 43–106; GF, 73 ± 14, 45–110; DABP: GT, 59 ± 11, 40–90; GF, 61 ± 14, 34–128 mmHg). The majority of cases of hypotension (7 of 10 versus 4 of 10 in the GF group) were observed during surgery until abdominal mammary removal. When the SABP was < 90 mmHg, it returned to satisfactory levels during anaesthesia once the isoflurane vaporiser setting was reduced and the fluid infusion rate was increased.

The ETIso concentration was 27% higher in the GF group (1.1 ± 0.2; 0.4–1.45) than in the GT group (0.8 ± 0.2; 0.1–1.2) (p = 0.0001). The ETIso values were higher in the GF group than in the GT group from the thoracic mammary gland divulsion (TM) to the end of surgery (ES). With the exception of the initial bolus, most animals in the GF group required fentanyl rescue during surgery (14 fentanyl boluses in 8 of 10 animals), mainly at the TM and AM time points, while no animals in the GT group required fentanyl rescue. There are no data regarding the possible isoflurane-sparing effect of lidocaine infiltration in dogs; however, the isoflurane-sparing effect of IV lidocaine has been previously reported in cats [29] and dogs [30,31]. Considering that the plasma lidocaine concentration in our study was similar to that achieved after IV infusion, it may be assumed that TA produces isoflurane-sparing effects as well. However, this topic warrants further investigation.

Steagall et al. (2006) [31] reported that the use of a fentanyl bolus (5 mcg/kg) followed by continuous infusion (0.5 mcg/kg/minute) provided sufficient analgesia and cardiovascular stability during mastectomy in bitches. Short-action opioid agonists, such as fentanyl, usually produce respiratory depression [5]. However, there was no increase in ETCO_2_ or apnoea in animals in the GF group.

There were no differences in the CVP (GT: 8.5 ± 4.8 cmH_2_O, GF: 6.5 ± 4.7 cmH_2_O), SpO_2_ (GT: 99.7% ± 0.7%, GF: 100% ± 0%) or T (GT: 34.5°C ± 1.2°C, GF 34.4°C ± 1.2°C) between the two groups. Although acute pulmonary oedema is a reported complication after the use of TA in humans, this aspect does not seem to be important in dogs, probably because relatively smaller injected volumes are used in this species. Hypothermia (< 37°C) developed in all animals during anaesthesia. The possible causes and adverse effects of intraoperative hypothermia have been reported elsewhere [32]. Although more severe hypothermia might be expected in the GT group because of the infiltration of a cold tumescent solution [33], there were no differences between the groups, showing that the solution did not intensify hypothermia. Hypothermia was probably produced by the sedative and anaesthetics used as well as the time of anaesthesia, even when basic care was provided for maintenance of temperature, such as the use of an electric blanket during surgery. At 240 minutes after extubation, the temperature of all animals in the GT group was ≥ 37°C. At 360 minutes, the temperature of two animals in the GF group was still < 37°C.

The only difference in surgical times between the groups was the shorter mammary excision time in the GT group (19 minutes shorter, p = 0.0195) compared with the GF group (Table 1). This was a result of the mechanical effect generated by the large volumes of the infiltrated solution [16,21], generating a ‘divulsion-like’ effect under the mammary tissue and facilitating removal of the tissue in the GT group. However, because there was no difference in the total duration of surgery, TA does not appear to be superior to fentanyl in terms of the time to perform surgery.

**Table 1:** Means ± standard deviations of age, weight, and surgical and anaesthetic recovery times (in minutes otherwise stated) in bitches submitted to unilateral mastectomy under tumescent anaesthesia (GT) or IV fentanyl bolus (GF) (‡ difference between groups)

**Groups**			
	**GT**	**GF**	**P value**
Age (years)	11.1 ± 2.16	9.7 ± 2.33	0.088
Weight (kg)	14.0 ± 10.43	13.14 ± 8.60	0.79
Total surgery time	77 ± 28	92 ± 28	0.25
Mammary gland excision time	30 ± 22	49 ± 19	0.02‡
Suture time	47 ± 11	43 ± 12	0.51
Time to extubation	6 ± 3	6 ± 3	0.88
Time to raising head	16 ± 21	7 ± 5	0.22
Time to sternal recumbence	45 ± 41	23 ± 18	0.15
Time to standing	105 ± 80	68 ± 51	0.23
Time for removal of the suture (days)	13 ± 1	14 ± 3	0.68

**Figure 2:**
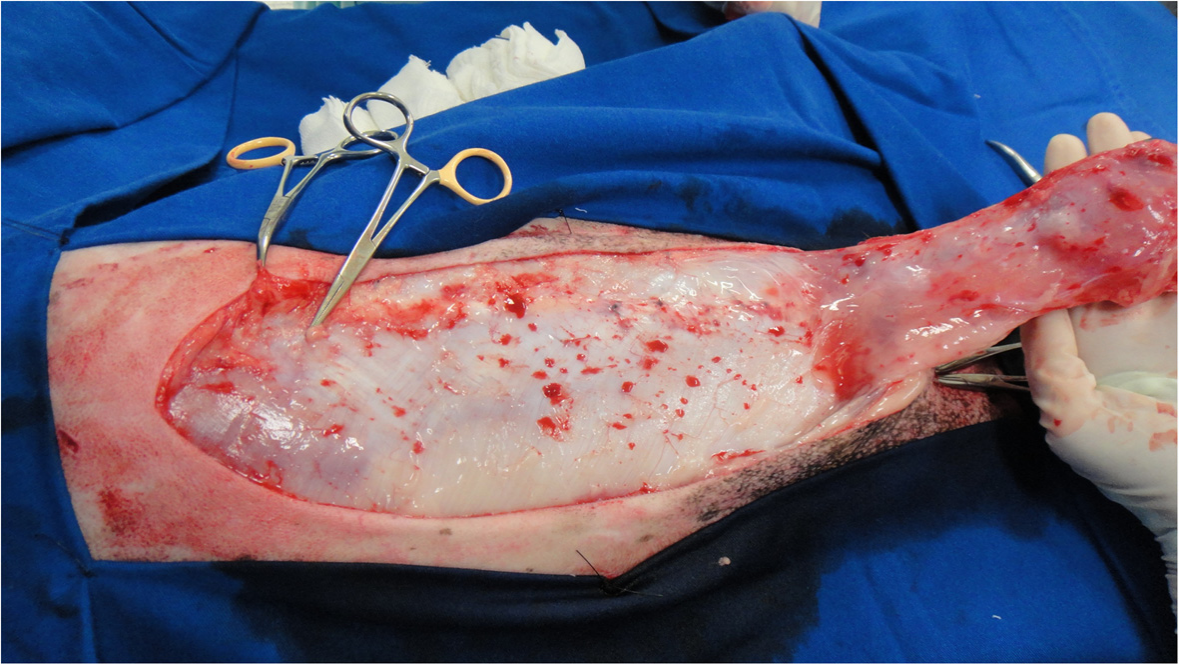
Reduced bleeding during mastectomy in one animal submitted to tumescent anaesthesia.

**Figure 3:**
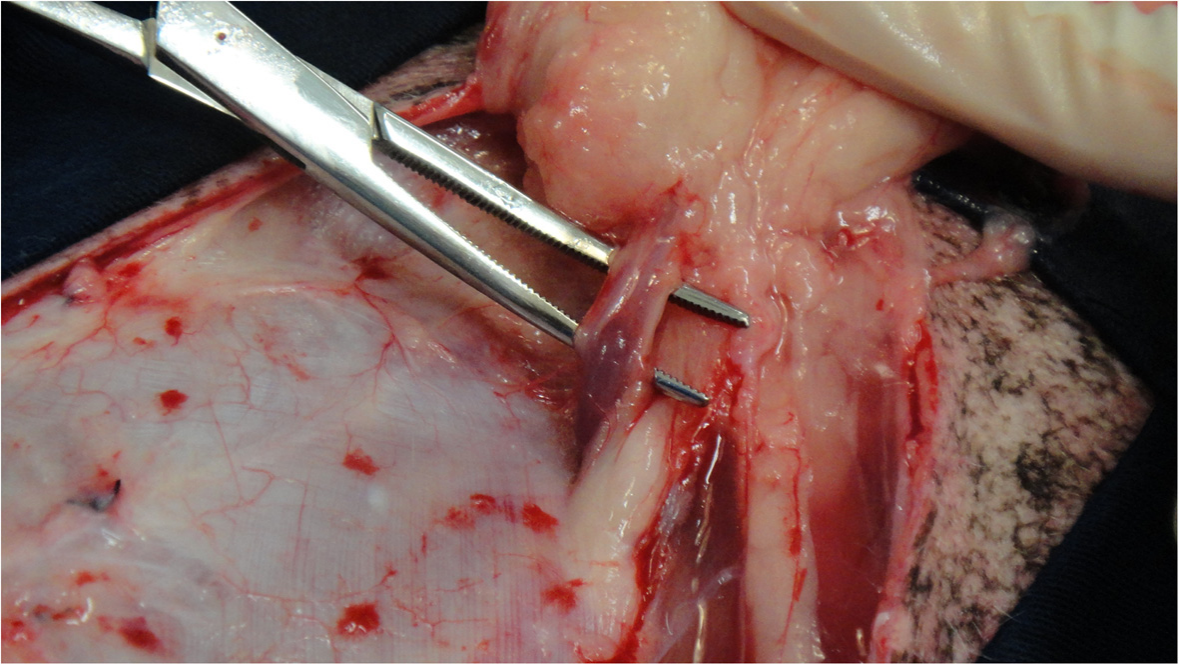
Clear visibilization of the pudendal vessels during mastectomy in one animal submitted to tumescent anaesthesia.

**Figure 4:**
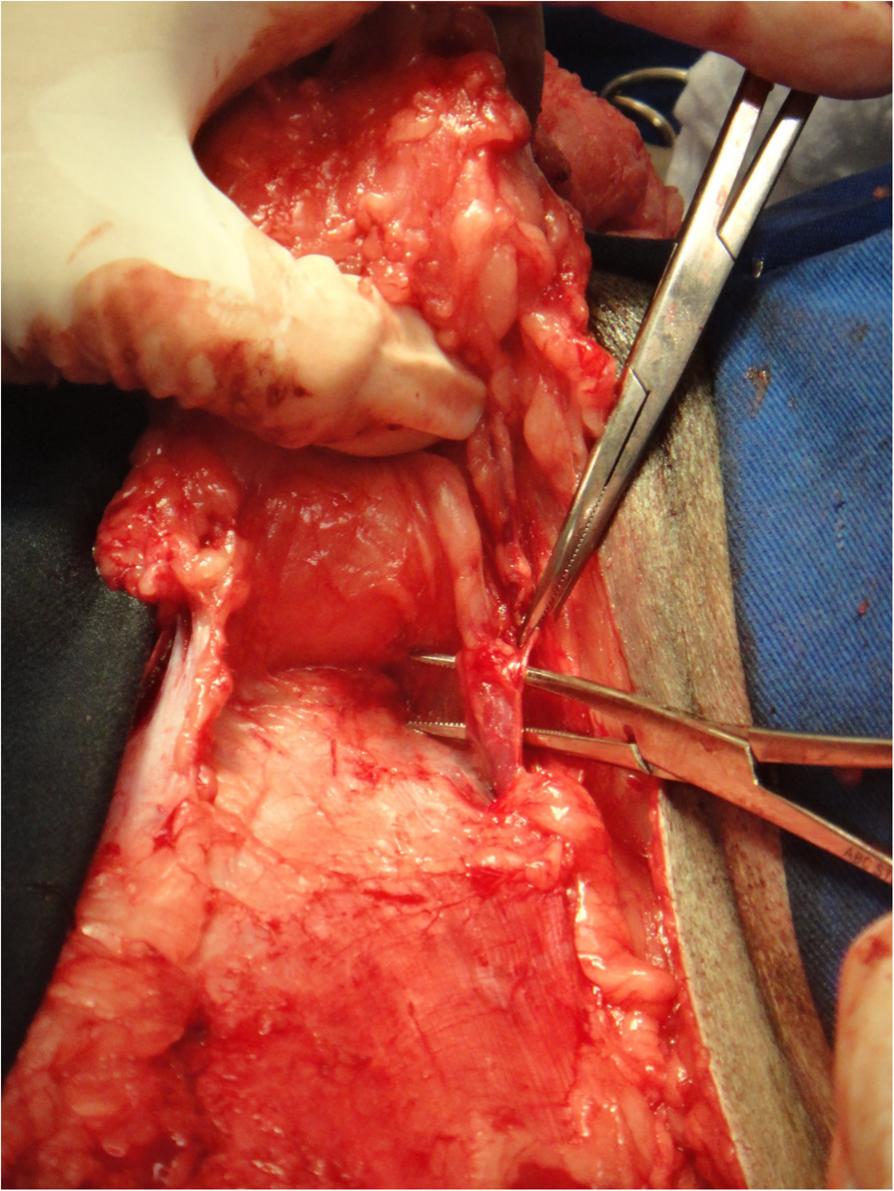
Visibilization of the pudendal vessels during mastectomy in one animal submitted to unilateral mastectomy without tumescent anaesthesia.

**Figure 5:**
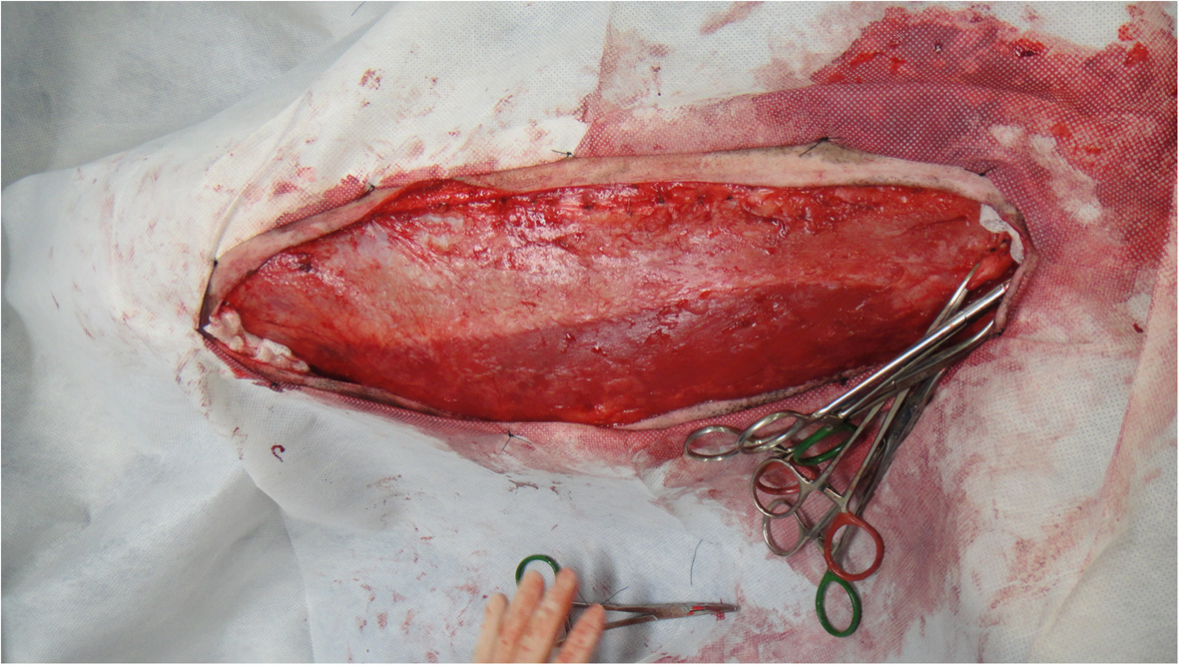
Bleeding during mastectomy in one animal submitted to unilateral mastectomy without tumescent anaesthesia.

The intraoperative bleeding was smaller in the GT group than in the GF group (p = 0.0012), as previously reported in TA. This is one of the main advantages of the TA technique, which is beneficial for liposuction in humans [14,17] and extensive skin [21,22] and oncological surgeries [16,20] (Figures 2 and 3). Adrenaline produces vasoconstriction, and the large infiltrated volume of the solution below the target tissue increases the hydrostatic pressure on small vessels, minimising bleeding [14]. Subjective evaluation of intraoperative bleeding was chosen here because objective evaluations, such as gauze-weighting, would suffer interference due to the large amount of fluid infiltrated into the tissue when the tumescent technique is used. This reduced bleeding volume also contributed to the shorter mammary excision time in the GT group, as previously described. When surgical bleeding is reduced, better visualisation of the operative area is allowed, and less vessel clamping (Figures 2, 3, 4 and 5), tissue manipulation, and suture material are necessary. It is widely known that intraoperative bleeding can aggravate the postoperative prognosis, especially in old patients and patients with systemic diseases. Bleeding impairs tissue oxygenation and, in oncological patients, is an important factor for tumour cell dissemination [34] and immunosuppression [35]. Although there was no significant difference, seven animals in the GF group and three in the GT group developed postoperative seromas that resolved 5 days after surgery. This demonstrates another potential surgical advantage of TA.

There were no wound-healing problems or dehiscence in any animal. Skin sutures were removed when the healing process was completed (10–14 days after surgery) [36].

**Figure 6:**
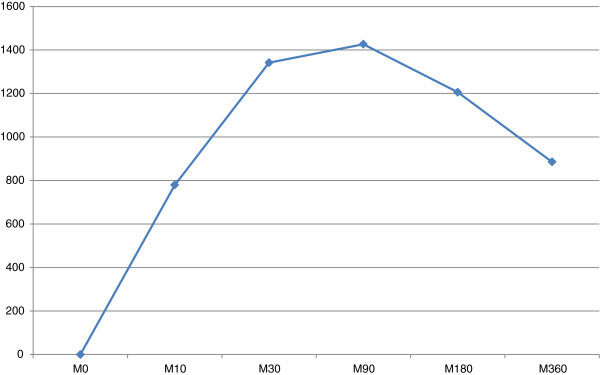
Mean plasma lidocaine concentrations after tumescent anaesthesia in bitches submitted to mastectomy (M0-M360: minutes after tumescent anaesthesia).

The maximal mean plasma lidocaine concentration was 1426 ± 502 ng/ml at 90 minutes after infiltration. The maximum individual plasma lidocaine concentration (2443 ng/ml) was observed at 90 minutes after infiltration. Peaks were achieved in five animals at 30 minutes, in four at 90 minutes, and in one at 180 minutes. The lowest concentration was 455.2 ng/ml at 10 minutes. In all cases except one, peak lidocaine plasma concentrations were achieved between 5 and 144 minutes after complete mammary gland removal, showing that systemic distribution has already occurred and biotransformation is relevant when TA is performed (Table 2 and Figure 6). Based on these findings, TA should be cautiously performed in animals with liver disease.

**Table 2:** Individual and mean (standard deviations) plasma lidocaine concentrations (ng/ml) after tumescent anaesthesia in bitches submitted to mastectomy

**Animal**	**M0**	**M10**	**M30**	**M90**	**M180**	**M360**
1	0	546.9	576.9	667.1	910.7	593.5
2	0	1148.7	2046.3	1653.8	1425.6	544.6
3	0	824.0	1496.9	1439.9	1109.0	740.8
4	0	651.9	1923.7	2443.0	1669.1	1376.1
5	0	817.6	1792.5	1603.7	1552.0	1263.9
6	0	1226.6	1291.2	1972.9	1476.9	1092.1
7	0	464.4	715.4	1220.3	1030.7	980.6
8	0	628.3	1697.7	1391.5	1273.2	964.5
9	0	1028.2	1414.1	852.0	938.3	822.8
10	0	455.2	463.2	1022.7	672.7	475.9
*Mean*	*0*	*779.18*	*1341.79*	*1426.69*	*1205.82*	*885.48*
*SD*	*0*	*278.73*	*572.13*	*502.85*	*323.82*	*304.77*

The single dose of IV lidocaine required to produce signs of toxicity (convulsion) in dogs is 22 ± 7 mg/kg [37]. Lethal doses range from 28 ± 1 to 76 ± 15 mg/kg when lidocaine is used as a single IV bolus or cumulative doses, respectively [38]. Several factors influence local anaesthetic toxicity, such as the time for absorption according to the local vascularisation, the route and site of administration, the local anaesthetic concentration, and association with a vasoconstrictor [39]. A bolus infusion of 4 mg/kg followed by a continuous infusion rate of 0.3 mg/kg/min of lidocaine induced muscular tremors after 24 ± 13 minutes. At this point, the plasma lidocaine concentration was 2.7 ± 1.1 mcg/ml [40]. In our study, there were no apparent signs of toxicity, even in the animal with the highest lidocaine concentration, which was still below the previously reported toxic concentration after IV lidocaine infusion [40]. The inclusion of the vasoconstrictor and the low lidocaine concentration in the tumescent solution (0.275%) probably contributed to the apparent lack of toxicity. Another important factor is that when the mammary tissue is removed, part of the anaesthetic solution is also removed, reducing further absorption of the local anaesthetic.

Although a considerable dose of lidocaine was used in our study (41.25 mg/kg), a previous study involving 60 human patients who underwent liposuction used up to 76.7 mg/kg of lidocaine, resulting in a plasma concentration of 3.6 mcg/ml. The authors concluded that lidocaine was safe at these dosages when used in low concentrations (0.05%–0.1%) [11].

The analgesic effect produced by lidocaine has been well recognised in several clinical and experimental models. Lidocaine attenuates neuropathic pain in rodents [41], produces thermal antinociception in horses [42], and provides perioperative analgesia in humans [43] and dogs [44]. Several mechanisms have been proposed to elucidate the analgesic effects of lidocaine and may be found elsewhere [45].

**Figure 7:**
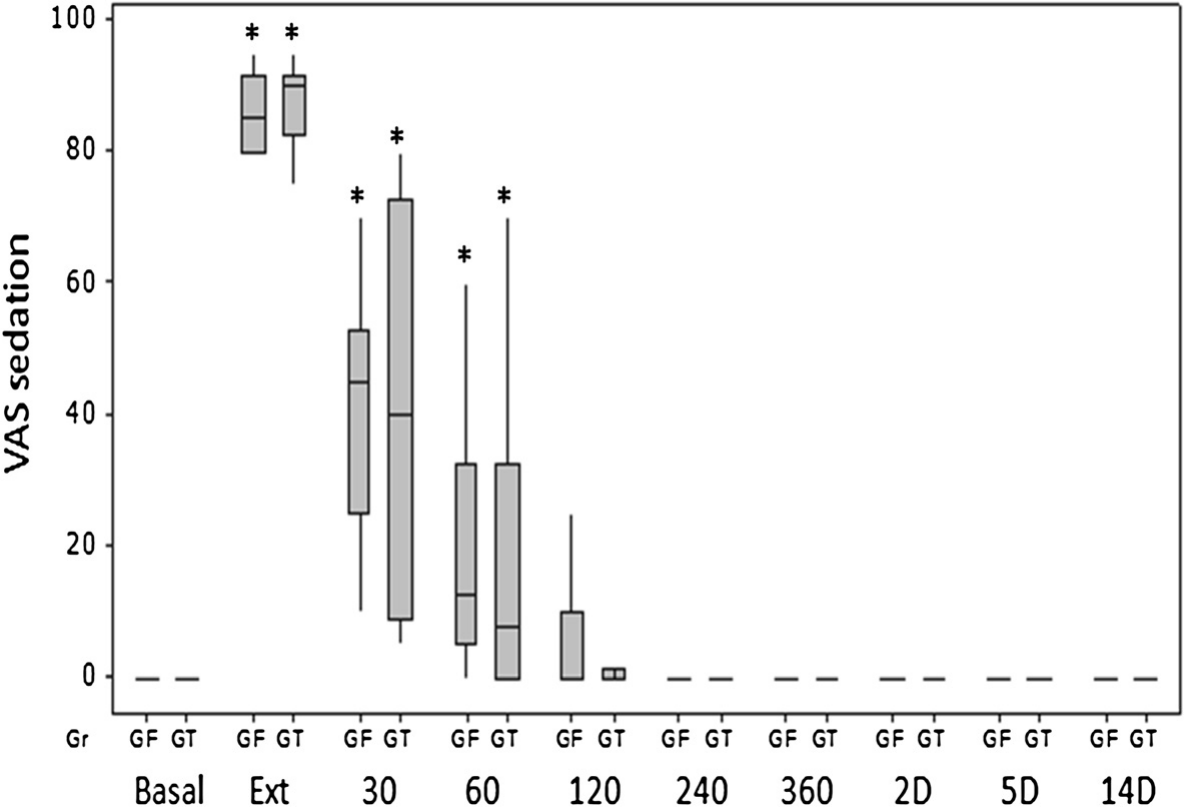
**Box plots of medians, 1st and 3rd quartiles of postoperative VAS for sedation after tumescent anaesthesia (GT) or IV fentanyl bolus (GF) in bitches submitted to mastectomy.** Legend: * Differences from basal in each group. Basal – before anaesthesia; Ext – at extubation; 30–360, minutes after extubation; 2-14D, days after surgery.

In this report, there was no difference in the number of morphine analgesic rescues (five in the GT group and six in the GF group) or the sedation scores between the groups. No postoperative rescue analgesia was performed when sedation was above 50% according to the VAS because of the major interference of sedation with the GCPS score. This was only observed at the first postoperative hour, at which time the scores were greater than the pre-anaesthetic basal values (p < 0.001) (Figure 7).

**Figure 8:**
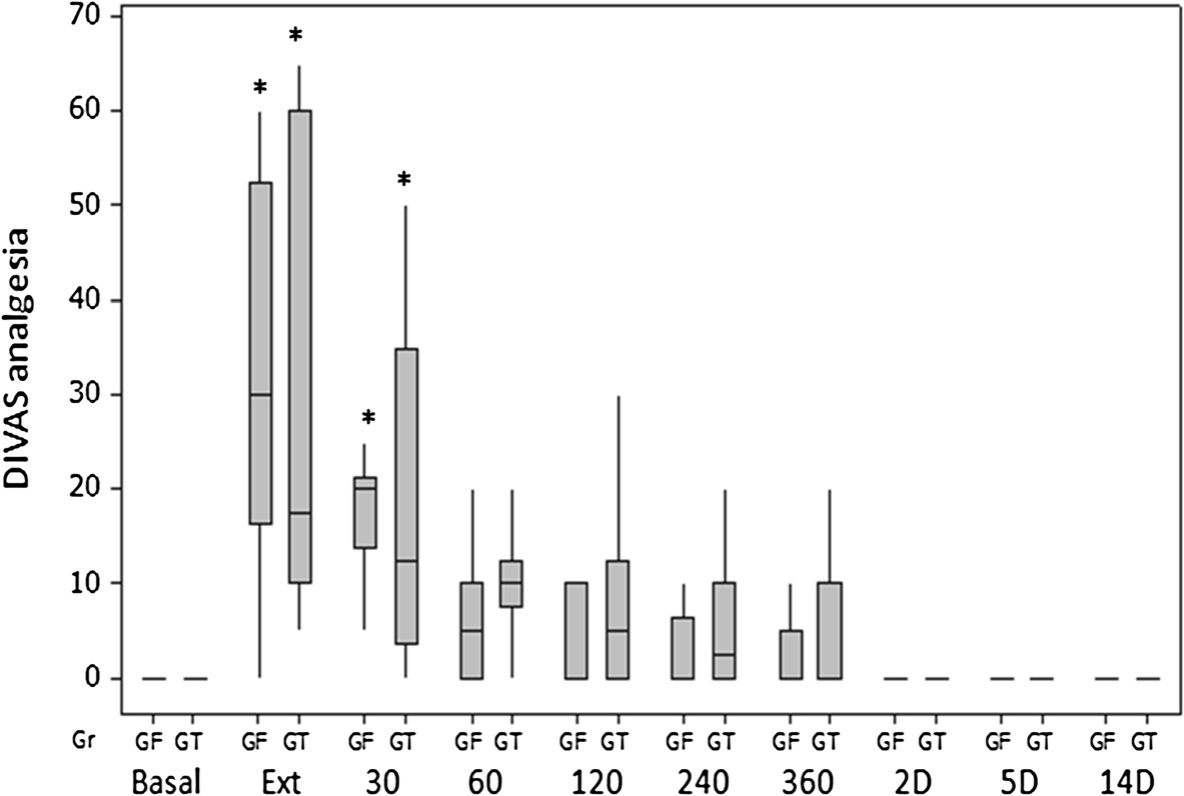
**Box plots of medians, 1st and 3rd quartiles of postoperative DIVAS for analgesia after tumescent anaesthesia (GT) or IV fentanyl bolus (GF) in bitches submitted to mastectomy.** Legend: * Differences from basal in each group. Basal – before anaesthesia; Ext – at extubation; 30–360, minutes after extubation; 2-14D, days after surgery.

The DIVAS pain scores were higher than the corresponding basal values from extubation until 30 minutes after extubation in both groups (Figure 8).

**Figure 9:**
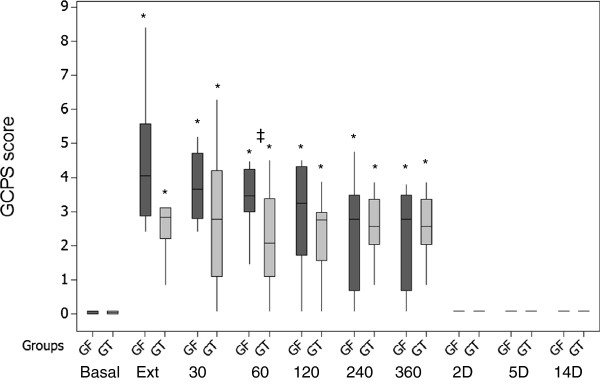
**Box plots of medians, 1st and 3rd quartiles of postoperative GCPS scores after tumescent anaesthesia (GT) or IV fentanyl bolus (GF) in bitches submitted to mastectomy.** Legend: * Differences from basal in each group; ‡ differences between groups at each time point. Basal – before anaesthesia; Ext – at extubation; 30–360, minutes after extubation; 2-14D, days after surgery.

**Figure 10:**
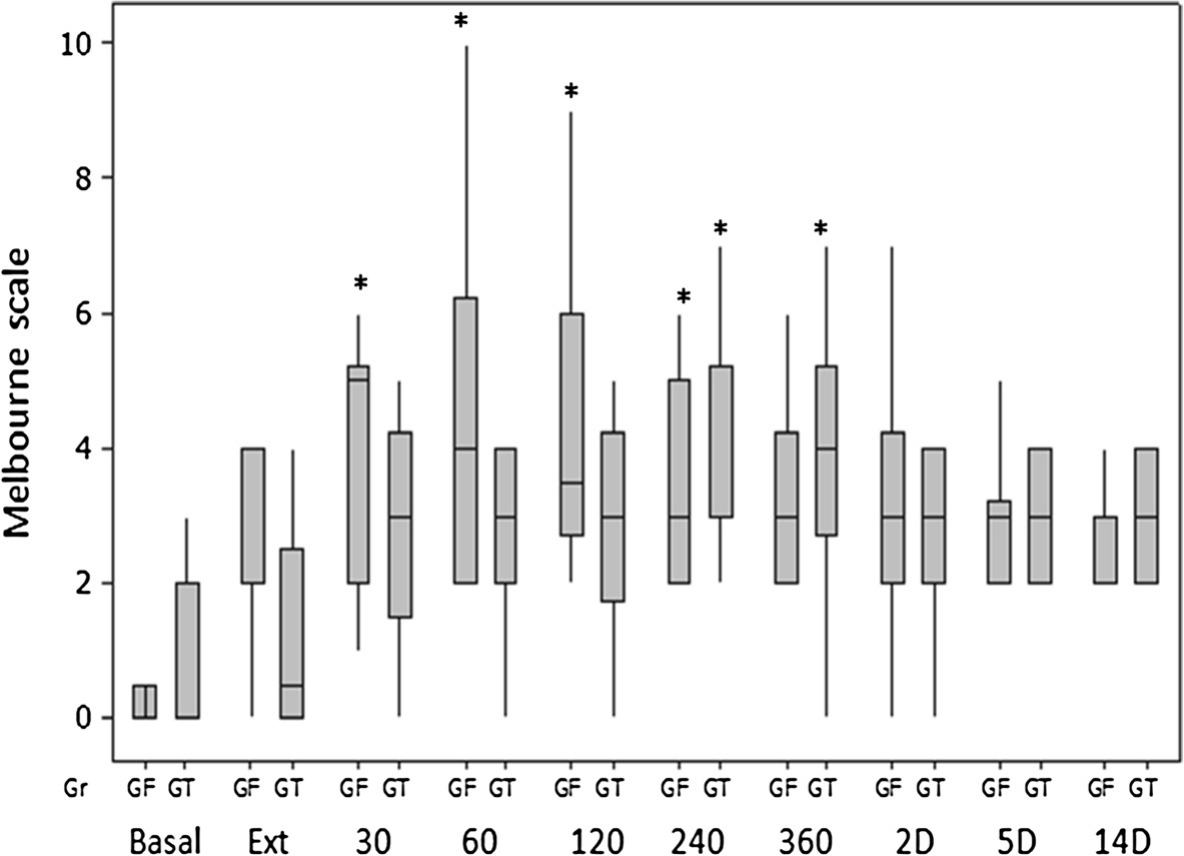
**Box plots of medians, 1st and 3rd quartiles of postoperative UMPS after tumescent anaesthesia (GT) or IV fentanyl bolus (GF) in bitches submitted to mastectomy.** Legend: * Differences from basal in each group. Basal – before anaesthesia; Ext – at extubation; 30–360, minutes after extubation; 2-14D, days after surgery.

The greater GCPS scores in GF at 60 minutes after extubation (p = 0.0494) and the earlier increased UMPS scores in the GF group (from 30 minutes after extubation until 360 minutes) than in the GT group (from 240 to 360 minutes after extubation), compared with the basal values, suggests that better postoperative analgesia was attained with TA in the bitches undergoing unilateral mastectomy in the present study (Figures 9 and 10), however a greater number of animals would be necessary to increase the power of the statistical tests and confirm that a better postoperative analgesia would be provided by TA. Most of the analgesic rescues were performed 60 minutes after extubation in the GF group (five of six in the GF group versus two of five in the GT group). This may be explained by the short postoperative analgesic effect of fentanyl [28].

The weights of three animals that received analgesic rescue in the GT group were <10 kg. Low-weight animals receive less total volumes of tumescent solution compared with heavier animals. In these cases, the volume might not be sufficient to cover the complete operated area in some breeds, such as Dachshunds. Thus, further dilution of the tumescent solution should be considered under these circumstances to increase the volume. However, analgesia may not be satisfactory in such cases.

Intravenous lidocaine infusion reduced the postoperative morphine consumption in dogs that underwent intraocular surgery [44]. Although the plasma lidocaine concentration was 0.4 mcg/ml during anaesthesia, the authors suggested that lidocaine induced preventive analgesia because the drug was undetectable in the plasma 2 hours after surgery and only 50% of the patients received analgesic rescue.

Lidocaine-induced postoperative analgesia is apparently observed when the plasma lidocaine concentration ranges from 0.4 to 1 mcg/ml in dogs [44] and from 1 to 3.7 mcg/ml in humans [46,47], providing better postoperative outcomes and shorter hospitalisation times [48].

Attenuation of the inflammatory response is the apparent mechanism behind lidocaine-induced analgesia [49]; however, there was no difference between the two groups for the Von Frey filament test (Table 3), suggesting that at least in this study, either hyperalgesia was not affected by TA or the number of animals was not sufficient to show differences between the groups.

**Table 3:** Median and semi-amplitude of Von Frey filament tests (grams/force) in bitches submitted to unilateral mastectomy under tumescent anaesthesia (GT) or IV fentanyl bolus (GF)

**Moments**	**Group**	
	**GT**	**GF**
Basal	300 ± 0	300 ± 0
Extubation	300 ± 0	300,± 145
30 min	300 ± 145	300 ±145
60 min	300 ± 145	10 ± 145
120 min	155 ± 145	10 ± 145
240 min	155 ± 145	10 ± 148
360 min	300 ± 145	10 ± 148
Day 2	300 ± 145	300 ± 145
Day 5	300 ± 0	300 ± 145
Day 14	300 ± 0	300 ± 0

Histopathological examination was performed in 9 of 10 animals in the GT group and in 10 animals in the GF group. Malignant neoplasia was found in six and seven animals from the GT and GF groups, respectively.

Although a short follow-up time elapsed after surgery (7–17 months), there were no tumour recurrences in any animal. This finding is beyond the scope of this study. However, it is well documented that regional anaesthesia attenuates the surgically induced endocrine responses and minimises or spares the use of opioids, improving the immunological response [50]. A reduction in opioid consumption was observed during the intraoperative period in the present study. Fentanyl and morphine depress natural killer cell activity and stimulate angiogenesis, cell migration, and cell proliferation in patients with cancer and therefore increases the occurrence of metastasis [6,9]. Thus, pure agonist opioids should be avoided in patients undergoing oncologic surgeries [7,51].

In this respect, regional anaesthesia is preferable to the use of general anaesthesia or opioids because it has been shown to reduce tumour recurrence in humans and rodents [52-54]. Lidocaine also inhibits epidermal growth factor receptor activity [34,55].

Considering that regional anaesthesia improved perioperative analgesia and surgical conditions in this study, and taking into account the potential effect of this technique in the postoperative prognosis of oncological patients, TA appears to be a promising technique under these circumstances.

## Conclusions

In conclusion, compared with IV fentanyl, the use of TA in bitches undergoing unilateral mastectomy: may be easily performed in noninflamed, ulcerated, or adhered mammary tumours; produces an isoflurane-sparing effect; improves transoperative and immediate postoperative analgesia; is apparently safe for use in clinical conditions as evidenced by the fact that it did not produce any adverse signs or lidocaine plasma concentrations compatible with toxicity; does not modify the recovery time; and facilitates the surgical procedure because of reduced bleeding and a shorter mammary gland removal time without interfering with wound healing.

## Endnotes

^a^Acepran 0.2%- Univet S/A- Veterinary Industry- Sao Paulo- SP, Brazil.

^b^Dolosal- Cristalia Chemicals and Pharmaceuticals Ltda- Itapira- SP, Brazil.

^c^Provive 1%-Claris Lifescience Limited- Sao Paulo- SP, Brazil.

^d^Isoforine- Cristalia Chemicals and Pharmaceuticals Ltda- Itapira- SP, Brazil.

^e^Isoflurane calibrated vaporizer 20318- HB Hospitalar Industria e Comercio Ltda- Sao Paulo- SP, Brazil.

^f^Fentanest- Cristalia Chemicals and Pharmaceuticals Ltda- Itapira- SP, Brazil.

^g^IntelliVue MP20, Philips Electronics, USA.

^h^Dimorf- Cristalia Chemicals and Pharmaceuticals Ltda- Itapira- SP, Brazil.

## Competing interests

The authors declare that they have no competing interests.

## Authors’ contributions

LFGAC participated in the design of the study, performed the tumescent anaesthesias, collected and organized the data, was responsible for anaesthesia until the beginning of surgery and draft the manuscript. SPLL conceived and coordinated the study, supervised data analysis, translated the manuscript and prepared the final draft. FF idealized the tumescent anaesthesia technique in dogs, participated in the design of the study and supervised the practical work. LCBAS helped in the design and setting up of the study, contributing to the collection of data. GBG performed the anaesthetic procedures (blind) and blind assessment of postoperative pain. JNNG performed all surgeries and contributed with technical advices. LRC performed and interpreted the statistical analysis and performed the figures. All authors read and approved the final manuscript.

## Authors’ information

All authors are Veterinarians, except LRC who is a mathematician. This study was a postgraduate project of LFGAC, who graduated in 2004, performed a Residence program in Veterinary Anaesthesiology in Guarulhos University in 2005–2007 and finished his Master of Science program at the Faculty of Medicine, from the University of Sao Paulo State (UNESP), Botucatu, Sao Paulo, Brazil in 2013. LFGAC was supervised by Professor SPLL and co-supervised by FF. SPLL is Full Professor of Veterinary Anaesthesiology at the Faculty of Veterinary Medicine and Animal Science, from the University of Sao Paulo State (UNESP), Botucatu, Sao Paulo, Brazil, where he has been working as a lecturer since 1987. SPLL got his PhD from University of Cambridge, England, in 1990 and since then has always been involved with research in anaesthesia, stress and pain. SPLL is a Diplomate from the European College of Veterinary Anaesthesia and Analgesia since 1995. He was Executive Director of the International Veterinary Academy of Pain Management between 2004 and 2008. FF got his graduation in 1999, Msci in 1998 and PhD in 2002, from the Department of Surgery, of the Faculty of Veterinary Medicine and Animal Science, from the University of Sao Paulo (USP), Sao Paulo, Brazil. He is currently an Associate Professor of Paulista University and Guarulhos University. LCBAS and GBG graduated in 2010 and performed a Residence program in Veterinary Anaesthesiology between 2011 and 2013, at the Guarulhos University. JNNG graduated in 2010 and completed her Residence Program in Small Animal Surgery in 2013, at Guarulhos University. LRC graduated in Mathematics in 1981, performed a Master of Health Profession Education at the University of Illinois – System, USA in 2003, Master in Agronomic Experimentation and Statistics at the University of Sao Paulo (USP), Sao Paulo, Brazil, in 1989 and PhD at the University of Sao Paulo State (UNESP), Botucatu, Sao Paulo, Brazil in 1996. She is currently the Head of the Biostatistical Department and Associate Professor University of Sao Paulo State (UNESP), Botucatu, Sao Paulo, Brazil.

## Supplementary Material

Additional file 1: Video 1 Tumescent anaesthesia in a dog before mastectomy. Click here for file
